# Caries Severity, Decayed First Permanent Molars and Associated Factors in 6–7 Years Old Schoolchildren Living in Palermo (Southern Italy)

**DOI:** 10.3390/jcm12134343

**Published:** 2023-06-28

**Authors:** Giuseppe Pizzo, Domenica Matranga, Laura Maniscalco, Fortunato Buttacavoli, Guglielmo Campus, Giovanna Giuliana

**Affiliations:** 1 Department of Surgical, Oncological and Oral Sciences, University of Palermo, 90127 Palermo, Italy; fortunato.buttacavoli@unipa.it (F.B.); giovanna.giuliana@unipa.it (G.G.); 2 Department of Health Promotion, Mother and Child Care, Internal Medicine and Medical Specialties, University of Palermo, 90127 Palermo, Italy; domenica.matranga@unipa.it (D.M.); laura.maniscalco04@unipa.it (L.M.); 3 Department of Restorative, Preventive and Paediatric Dentistry, University of Bern, 3012 Bern, Switzerland; guglielmo.campus@unibe.ch; 4 Department of Surgery, Microsurgery and Medicine Sciences, School of Dentistry, University of Sassari, 07100 Sassari, Italy

**Keywords:** dental caries, caries prevalence, caries severity, children, socio-economic condition, socio-economic determinants, socio-economic disparities in health, Italy

## Abstract

To date, there are very few epidemiologic studies on caries disease in 6–7 year old children living in Sicily (Southern Italy). The first permanent molar (FPM) is the most commonly affected tooth in this target population, and a one-unit increase in the number of decayed FPMs is predictive of caries in other teeth and in adulthood. The primary aim of this research is to estimate the prevalence of caries in 6–7 year old schoolchildren living in Palermo and, as a secondary aim, to estimate the prevalence of affected FPMs. It was designed as a cluster cross-sectional survey on 995 children from 16 schools, selected based on their geographical location, in one of the eight city districts. Caries data were recorded using the International Caries Detection and Assessment System for each tooth surface. The relation between socio-economic status, behavioural determinants, and clinical information and the number of teeth with initial caries (IC), moderate caries (MC), or extensive caries (SC) was analysed through the ordinal logistic regression. Among the 995 schoolchildren, 662 (66.5%) had at least one lesion and 742 (74.6%) had FPMs. Of the latter, 238 (32.0%) were affected by IC, 86 (11.6%) were affected by MC, and only 3 (0.4%) were affected by SC. During multivariable analysis, there was evidence of an increased risk of MC and SC related to the deprivation of the district in which the children lived and went to school, as well as to the protective role of parental education and employment. The same significant determinants were found for IC and MC FPMs. The study showed the important role of socio-economic determinants, unhealthy behaviours, and social deprivation related to the increased risk of moderate and extensive caries in 6–7 year old schoolchildren. Investigating this target population is very important, as early development of caries in FPMs may have serious consequences in the prognostics of oral health in an adult.

## 1. Introduction

In Western countries, dental caries are serious public health concerns, despite the efforts that have been made to reduce their prevalence among children and adolescents. Based on a recent systematic review and meta-analysis, the overall prevalence of dental caries in children ranges between 46.2% for primary teeth and 53.8% for permanent teeth, with the highest percentages observed in Africa and Asia and the lowest ones observed in Europe [1]. Oral epidemiology research has not been a major focus of Italian researchers or funding agencies compared to other areas of study. Moreover, the Italian public health sector has been undergoing a lengthy period of change and scrutiny, with a significant decrease in the proportion of Gross National Product (GNP) spent on health care and, especially, oral health care. In Italy, few epidemiological surveys have been conducted on the prevalence of caries, and these generally showed a marked decrease in caries prevalence over time [2,3,4]. However, to date, there are very few studies on the oral health of children living in Southern Italy and, particularly, in Sicily [5,6]. Sicily might be considered a cluster, as the region has peculiar socio-economic features (low socio-economic indicators at individual levels, as well as negative macroeconomic indicators). Caries, especially in disadvantaged groups, remain huge public health problems around the world with an increasing burden of untreated caries. A high caries prevalence in young children living in Italy, especially in those with non-European backgrounds, was recently described [3]. Quite strong differences between Northern and Southern Italy were observed, and severe lesions with cavitation were more prevalent in children living in the Southern than in the Northern and Central Italy. Both macroeconomic indicators (GNP per capita, Gini Index, and Unemployment rate) and individual socio-economic indicators (nationality, educational level, working, status and smoking habits) were significantly correlated to the prevalence and severity of caries, as well as to the prevalence of restorations due to caries [2,3].

There is evidence that children with high caries experience show an increased risk of caries in adulthood [7]. The first permanent molar (FPM) is the most common tooth affected by dental caries in 6–7 years olds [8], and a one unit increase in the number of carious first molars is associated with a significant increase in the number of other carious teeth [9]. Therefore, FPMs are very important not only because of their significant role in maintaining a normal masticatory function and dental–facial harmony but also as predictors of caries in other teeth and in adulthood [8,9,10]. 

The role of socio-economic context in the epidemiology of dental caries is quite noteworthy. Children from low-income households show a higher caries prevalence and more severe clinical manifestation than those from high-income households [3,11]. The educational level and the socio-economic status of parents significantly influence their children’s oral health status [2,12,13] due to poorer knowledge concerning oral health, including different factors, such dietary habits and daily sugar intake or oral hygiene habits, as well as reduced access to dental services [14]. The macro-context of the birth place and residential location of the child also had to be considered due to the association between the social position of a person and the risk of poorer health and reduced access to health care services [15]. Social class, socio-economic position, and the geographical location and stratification within the city can be considered as notable predictors of the disease rate with deeper interconnections, so it is crucial to understand the social inequalities in oral health to improve the health of the whole population [7,16].

Epidemiological data collection on carious lesions is usually carried out through the direct visual detection of lesions by trained examiners. The actual data on caries are based on the International Caries Detection and Assessment System (ICDAS), which is a simple evidence-based method for caries detection that is capable of classifying caries severity by the stage of carious lesions [17,18]. The WHO method for caries detection, widely used until the last decade, could not adequately assess the prevalence and severity of caries since different stages of caries are not assessed [10,19]. In geographical areas with low prevalence of the disease, planning effective strategies for caries prevention and control results in failure. Moreover, the early detection of carious lesions at non-cavitated levels is now considered to be a key target in the overall effort to move away from operative and towards non-operative preventive dentistry [20]. 

Starting from these premises, a cross-sectional investigation was planned and carried out that aimed to elucidate the caries experience of 6–7 years old children living in Palermo (Sicily, Southern Italy). The focus was on demographic, socio-economic, and behavioural characteristics that are relevant for discriminating between moderate and extensive lesions, as measured through the ICDAS. Furthermore, the prevalence of FPMs affected by caries was investigated as a prognostic indicator of oral health in adulthood. 

## 2. Materials and Methods

### 2.1. Study Design and Sample Selection

The population objects of the survey were the children (6–7 years of age) living in the Palermo area (Sicily, Southern Italy). It was designed as a cluster cross-sectional survey on children sampled from 16 schools, selected based on their geographical location in one of the eight city districts, according to the school headteachers’ willingness and availability to participate in the survey (response rate: 53.3%). The sample size was calculated while taking into account the literature data on caries prevalence in Italy [5]. The assumed frequency of the disease in the population (literature prevalence: 36%) was increased by 5% to protect the results from a possible increase in prevalence in the study area, and the confidence level was set at 99%. Assuming an average number of children per class of 25 children, the needed sample size was 612 subjects, which was increased to 765 children (25% more) to estimate a rate of non-responders. The sample size for each district was determined using the data from the 2011 national census population (The Italian National Institute of Statistics; ISTAT). Data collection was carried out from February 2017 to February 2019.

The survey was planned according to the protocol of the second national pathfinder, conducted in Italy, on children’s oral health from November 2016 to June 2017. The original survey protocol was approved by the Ethics Committee of the University of Sassari, Italy (AOUNIS: 29/16). An additional approval of the survey protocol was obtained from the Ethics Committee of the University Hospital “Policlinico Paolo Giaccone” in Palermo, Italy (approval number 2/2022). All parents/caregivers of the children were requested to sign informed consent forms. The survey was conducted according to the ethical guidelines of 1964 Declaration of Helsinki and its later amendments or comparable ethical standards. 

### 2.2. Data Collection and Variables

A standardised questionnaire [21] on family social background, mother’s and father’s education (categorised as middle school or lower vs. high school or higher), as well as mother’s and father’s working status (coded as employed vs. unemployed/homemaker) was filled-in by parents/caregivers. The questionnaire also included clinical information about children’s oral health and hygiene, such as daily toothbrushing habits (often vs. never/seldom), additional fluoride products used at home or applied by a professional (yes vs. no), dentist visits in the previous year (>1 vs. ≤1), as well as current or former wearing of orthodontic devices (yes vs. no). Finally, the questionnaire collected behavioural information, such as chewing gum consumption, daily fruit consumption, weekly dairy product consumption, using a sweetened pacifier when younger, eating snacks between meals, and drinking sweet/carbonated beverages, all categorised as often/always vs. seldom/never.

Data was collected by means of clinical examinations using a plain mirror (Hahnenkratt, Königsbach, Germany) and the WHO ballpoint probe (Asa-Dental, Milan, Italy) under artificial light. Caries data were recorded using the two-digit codes related to ICDAS for each tooth surface: each tooth was recorded as caries-free (ICDAS_0_), initial lesion (ICDAS_1–2_), moderate lesion (ICDAS_3–4_), extensive lesion (ICDAS_5–6_), filled teeth due to caries (Ft), and missing teeth due to caries (Mt) [2]. Clinical examinations of participants were carried out by a unique, previously calibrated rater (G.P.) [2]. The Italian Deprivation Index (DI) was considered at the city level, calculated at the census section level as the sum of standardised indicators of five 2011 national census variables, with these being the percentages of low educated people, unemployed people, rented housing families, one-parent families, and high-density housing [22].

### 2.3. Statistical Analysis

There were three response variables considered, as the number of teeth in the child’s mouth with initial caries (IC), moderate caries (MC), and extensive caries (EC). A tooth was defined with IC if the maximum ICDAS on its surfaces was 1 or 2. It was defined with MC if the maximum ICDAS on its surfaces was 3 or 4, while it was reported with SC if the maximum ICDAS on its surfaces was 5 or 6. The number of caries-free (CF) teeth was calculated as the number of teeth in the child’s mouth with ICDAS = 0 on all surfaces.

At univariable analysis, CF, IC, MC, and SC were synthesised as the mean (±standard deviation). Categorised responses were summarised as counts and percentages. Demographic, socio-economic, behavioural, and clinical characteristics were also expressed as counts and percentages. Deprivation (DI) was classified through the quintiles ranked from the least deprived (1st and 2nd quintiles) to the most deprived (3rd, 4th, and 5th quintiles). The difference of IC, MC, and SC by demographic, socio-economic, behavioural, and clinical characteristics was assessed through one-way ANOVA. The association among categorical variables was assessed using the chi-square test.

During multivariable analysis, the responses were recoded using three ordinal categories (0 teeth, 1 tooth, and ≥2 teeth, affected by IC, MC, or EC). Similar classification was proposed and limited to the first permanent molars. The stepwise ordinal logistic regression model was estimated by choosing a *p*-value equal to 0.01, to include and equal 0.05, to exclude variables.

Statistical analysis was performed using STATA/IC 15. The *p* < 0.05 was chosen to assess statistical significance.

## 3. Results

### 3.1. Sample Description 

Overall, 52.6% (*n* = 523) of the sample was male and more than 70% (*n* = 711) of the children were aged 6. Most of the children (*n* = 713; 71.7%) attended schools in the most deprived municipal districts (the 5th quintile of DI is the most frequent for the first, second, fifth, and sixth districts, the 4th quintile is for the seventh district, and the 3rd quintile is for the third district) (Table S1). There were 662 schoolchildren (66.5%) with at least one lesion, with no difference by sex (52% males). No restorations were found in 601 children (90.8%), whereas 60 subjects (9.1%) had at least one restoration; only one child (0.2%) had dental sealants (data not shown). 

### 3.2. Initial Lesions (ICDAS_1–2_) 

The mean number of teeth with initial caries lesions (IC teeth) was 1.02 ± 1.34. There were 498 (50.0%) children with at least one IC tooth (Table 1). Children with highly-educated mothers had lower ICDAS_1–2_ levels (0.93 ± 1.30) compared to children with less-educated mothers (1.13 ± 1.39) (*p* = 0.03). The same feature was also observed among children with employed mothers (0.92 ± 1.33) compared to those with unemployed/homemaker mothers (1.06 ± 1.35) (*p* = 0.022). There were significant differences in the average number of IC teeth among children from schools located in different city districts (*p* < 0.001). ICDAS_1–2_ levels for children from schools of the most deprived districts were significantly higher compared to the least deprived districts (*p* = 0.002) (Table S1). 

When clinical and behavioural characteristics were considered, a significant difference in ICDAS_1–2_ levels was found between children with at least one decayed first permanent molar (2.34 ± 1.47) compared to those without (0.54 ± 0.91) (*p* < 0.001) (Table S2). 

The multivariable analysis confirmed the increased risk of IC for children attending school in deprived districts (AdjOR: 1.46, 95% CI: 1.07–1.99, *p* = 0.017), particularly in the second, fifth, and seventh districts; a decreased risk for children attending schools in the third district was also found (Table 2).

### 3.3. Moderate Lesions (ICDAS_3–4_) 

The mean number of teeth with moderate caries lesions (MC teeth) was 1.03 ± 1.52. There were 434 (43.6%) children with at least one MC tooth (Table 1). As described for IC, children with less-educated mothers or fathers had high caries lesions (1.49 ± 1.7 and 1.39 ± 1.68, respectively) compared to children with highly-educated parents (mother 0.66 ± 1.23 or father 0.66 ± 1.23; *p* < 0.01). Similarly, the difference in the average number of ICDAS_3–4_ teeth was significantly higher for children with an unemployed/homemaker mother (1.22 ± 1.58) or father (1.5 ± 1.68) compared to employed mother (0.66 ± 1.29) or father (0.87 ± 1.42) (*p* < 0.001). The highest mean number of affected teeth per mouth was found for children attending a school in the first district (2.14 ± 1.72), while the lowest was recorded for schools in the eighth district (0.53 ± 1.1) (*p* < 0.001). The mean levels of ICDAS_3–4_, between children attending schools of the least and most deprived districts, were significantly different (0.56 ± 1.10 vs. 1.20 ± 1.61; *p* < 0.001) (Table S1).

With regards to behavioural characteristics, significant differences in ICDAS_3–4_ levels were found between children who brushed after each meal and those who did not (0.94 ± 1.46 vs. 1.32 ± 1.65; *p* = 0.006). Similar findings were found when the following variables were assessed: additional fluoride product use (0.8 ± 1.45 vs. 1.08 ± 1.52; *p* = 0.002), chewing gum consumption (1.74 ± 1.78 vs. 0.91 ± 1.44; *p* < 0.001), daily fruit consumption (1.38 ± 1.65 vs. 0.92 ± 1.45; *p* < 0.001), sugary drink/sweetened pacifier use before bedtime (1.59 ± 1.71 vs. 0.95 ± 1.47; *p* = 0.005), and sweet drink/carbonated drink consumption (1.32 ± 1.59 vs. 0.86 ± 1.44; *p* < 0.001). When clinical characteristics were considered, children visiting a dentist no more than twice in the previous year had better mean ICDAS_3–4_ levels (0.91 ± 1.43) compared to those who visited a dentist more than twice (1.87 ± 1.93) (*p* < 0.001). Moreover, there were significant differences in ICDAS_3–4_ levels between children with at least a decayed permanent first molar (1.81 ± 1.75) and those without (0.74 ± 1.31) (*p* < 0.001) (Table S2). 

Looking at multivariable analysis, having highly-educated parents plays a protective role on their children’s MC lesions (mother AdjOR: 0.64, 95% CI: 0.44–0.91; *p* = 0.014, and father AdjOr: 0.65, 95% CI: 0.46–0.93; *p* = 0.018). It was also confirmed that the increased risk of MC was associated with the frequent use of chewing gum (AdjOr: 1.61, 95% CI: 1.03–2.52, *p* = 0.038), consumption more than two pieces of fruit per day (AdjOR: 1.82, 95% CI: 1.3–2.55, *p* = 0.001), frequent consumption of sweet drinks/carbonated drinks (AdjOR: 1.63, 95% CI: 1.19–2.23, *p* = 0.002), and more than two visits to a dentist in the past year (AdjOR: 2.80, 95% CI: 1.75–4.48, *p* < 0.001). Lastly, children attending school in the first, second, fifth, sixth, and seventh districts of Palermo were at increased risk of higher levels of ICDAS_3–4_ (Table 2).

### 3.4. Extensive Lesions (ICDAS_5–6_) 

There were 244 (24.5%) children with at least one SC tooth. The mean number of SC teeth was 0.69 ± 1.61 (data not in tables). At univariable analysis, significant differences in ICDAS_5–6_ levels were found between children with a less-educated mother/father compared to those with a highly-educated mother/father (1.12 ± 2.05 vs. 0.34 ± 1.01 and 1.03 ± 1.92 vs. 0.31 ± 0.98, respectively; *p* < 0.001, in both cases). Similar findings were detected when their mothers’/fathers’ working status was assessed (0.86 ± 1.8 vs. 0.39 ± 1.13 and 1.25 ± 2.04 vs. 0.5 ± 1.35, respectively; *p* < 0.001, in both cases). The highest ICDAS_5–6_ levels were found for children attending school in the first district (2.14 ± 3.24), while the lowest was recorded in the fourth district (0.33 ± 0.93) (*p* < 0.001). ICDAS_5–6_ levels were significantly lower in children from schools of the least deprived districts compared to the most deprived ones (0.31 ± 1.16 versus 0.83 ± 1.72; *p* < 0.001) (Table S1).

With regards to behavioural characteristics, significant differences in ICDAS_5–6_ levels were found between children who brushed teeth after every meal compared to those who did not (0.64 ± 1.55 vs. 0.88 ± 1.79; *p* = 0.032). Similar findings were found when the following variables were assessed: chewing gum consumption (1.43 ± 2.35 vs. 0.59 ± 1.45; *p* < 0.001), daily fruit consumption (0.96 ± 1.97 vs. 0.58 ± 1.43; *p* = 0.008), dairy product consumption (0.93 ± 1.84 vs. 0.53 ± 1.41; *p* = 0.008), sugary drink/sweetened pacifier consumption/use before bedtime (1.88 ± 3.22 vs. 0.57 ± 1.36; *p* = 0.007), and sweet drink/carbonated drink consumption (1.07 ± 2.04 vs. 0.51 ± 1.33; *p* < 0.001) (Table S1). When clinical characteristics were considered, children visiting a dentist no more than twice in the previous year had better mean ICDAS_5–6_ levels compared to those that visited a dentist more than twice (0.57 ± 1.5 vs. 1.64 ± 1.93; *p* < 0.001). Children without carious lesions on their first permanent molars had better mean ICDAS_5–6_ levels than children with at least a decayed first permanent molar (0.47 ± 1.31 vs. 1.30 ± 2.11; *p* < 0.001) (Table S2).

During multivariable analysis, the protective role against the development of SC, from having a highly educated father (AdjOR: 0.44, 95% CI: 0.29–0.66, *p* < 0.001) and the frequent intake of dairy products (AdjOR: 0.63, 95% CI: 0.44–0.92, *p* = 0.015), was confirmed. On the other hand, there was an increased risk of SC for children whose fathers were unemployed/homemakers (AdjOR: 2.30, 95% CI: 1.52–3.47, *p* < 0.001), for children who frequently chewed gum (AdjOR: 2.25, 95% CI: 1.39–3.65, *p* = 0.001), and for those who visited a dentist more than twice a year (AdjOR: 7.12, 95% CI: 4.25–11.94, *p* < 0.001). Moreover, it was confirmed that there was increased risk for children attending schools located in the first district (AdjOR: 3.60, 95% CI: 1.37–9.41, *p* = 0.009), the second district (AdjOR: 1.99, 95% CI: 1.27–3.12, *p* = 0.003), and the seventh district (AdjOR: 1.82, 95% CI: 1.02–3.22, *p* < 0.041) (Table 2).

### 3.5. First Permanent Molars

Among the whole sample, 742 (74.6%) children had FPMs, and of these, 238 (32.0%) were affected by IC, 86 (11.6%) were affected by MC, and only 3 (0.4%) were affected by EC (Table 3).

The mean value of surfaces affected by IC on FPMs was 0.85 ± 1.49, and the occlusal surfaces were more frequently affected than the non-occlusal ones (0.54 ± 0.93 vs. 0.31 ± 0.63; *p* = 0.001). When MC was considered, the mean number of affected surfaces on FPMs (0.27 ± 0.88) was higher in the occlusal than in the non-occlusal ones (0.18 ± 0.56 vs. 0.09 ± 0.38; (*p* < 0.001). When SC was considered, the mean number of affected surfaces was negligible (0.01 ± 0.11) (Table 4). 

The risk of IC in the FPMs was found to be related to the school’s district (increased risk for those attending schools in the second, fifth, and seventh districts and decreased risk for the third and sixth districts), and it was higher in children with unemployed/homemaker mothers and children living in deprived districts. Similarly, the risk of MC in the FPMs was higher for schools in the sixth district and lower for children with highly-educated father. Data for SC is not shown because of insufficient cases (Table 5).

## 4. Discussion

The aim of this paper was to assess the oral health status of 6–7 year-old schoolchildren living in Palermo (Sicily, Southern Italy) while exploring the geographical differences among city districts and the role of socio-economic deprivation. A previous study of this research group did not show a significant role of deprivation to explain variability in dental caries, but this result was found on a different population target (12 years old) and by using the DMFT index [23]. Compared to the ICDAS-II method, the WHO method for caries detection underestimates caries prevalence. In fact, children with non-cavitated lesions, classified as caries-free following the WHO criteria, are indeed classified as ICDAS [1,23]. Consequently, the ICDAS method is also more sensitive in the accurate recording of the severity of carious lesions. Nowadays, the necessity of the early detection of carious lesions at the non-cavitated level is known as the key target in the overall effort to move away from operative and towards non-operative preventive dentistry [20].

**Figure 1:**
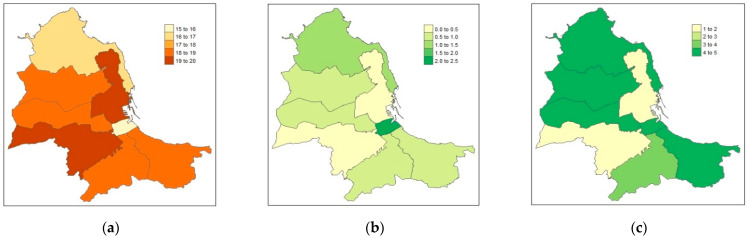
Spatial distribution of caries-free (ICDAS_0_) (**a**), extensive lesion (ICDAS_5–6_) (**b**), and the Deprivation Index (**c**) in the eight municipal districts of Palermo. The Deprivation Index ranges between 1 (the least deprived) and 5 (the most deprived).

An interesting result of the present study is that almost two-thirds of the children aged 6–7 years, living in Palermo, were found with at least one caries lesion. Secondly, children with the disease were unevenly distributed among city districts, with the highest prevalence of caries-free children in the least deprived districts and the highest prevalence of severe caries in the most deprived areas (Figure 1).

These alarming results reflect the evidence from a recent Italian survey of a higher risk of cavitated caries lesions for children living in low income and high unemployment rate geographical areas, albeit at a microlevel [2]. Healthcare in Italy is provided by a mixed public and private system, but oral healthcare is largely provided by private practitioners and is mainly financed by direct payment by the patient or, to a lesser extent, by private insurance schemes. In this situation, the role of socio-economic factors on oral health outcomes is significant, reflecting the knowledge and spending capacity at the individual level but contextualizing the reality in which families live at the population level. Palermo is the fifth Italian city by population size, and it is 1 of 15 Italian Metropolitan cities, which are characterised by low educational levels (38.8% of young people aged 15–29 neither study nor work, +16.3% than the national average), low employment rates (41% of the active population aged 15–64 was employed, −20.1% than the national average), and large swathes of poverty (the disposable income per capita was 13,687 euros less than the national average of 17,307 euros) [24]. Furthermore, the distribution pattern of caries staging in the local population reflects the socio-economic context of families living in differently deprived districts. Based on the most recent official statistics published on the website of the Municipality of Palermo, the first district reported the highest inter-census growth rate (+8.8%), the highest percentage of foreign residents per 100 inhabitants (20%), and the structural youth dependency index (25.2%) and elderly dependency index (141.5%) were greater than the city averages (22.2% and 83.8%). The second, sixth, and seventh districts also reported demographic and economic indicators that were lower than the city’s average. The survey data analysis is consistent with the literature using ICDAS, especially with regards to the protective role of highly educated parents [25] and the risk factor represented by a father’s unemployment, especially for SC [26]. In fact, the transmission of basic knowledge, for the prevention of dental caries, occurs during the developmental age through the parent who guides their children’s behaviour by taking inspiration from their own education. The data from the present survey also show the importance of low socio-economic indicators, attributable to the father (head of the family), for the oral health of children.

Unhealthy dietary habits, particularly the frequent consumption of sweet drinks/carbonated drinks, were found to be associated with a higher risk of MC, confirming what has been shown for 12-year olds children using the DMFT index [27]. The nutritional style is widely conditioned by socio-economic background, and the most disadvantaged population strata tend to have worse diets that are less rich in nutrients and composed of processed unhealthy products, such as sugared beverages (cola, fruit juices, or sweet syrups) and sweet snacks [28]. A diet rich in food and beverages containing sugar increases the risk of bacterial fermentation in dental plaque, resulting in caries. Moreover, the associations of socio-economic status and food security with children’s caries has been widely demonstrated [29,30].

In addition, the habit of chewing gum was found to be a risk factor for SC too. Bubble gum is very attractive for children because of its sweetness, colours, and noise. In Italy, some chewing gums sweetened with sugar alcohols are available; however, their xylitol content is generally low. Moreover, these products are advertised to be sold to an adult audience, rather than children. Sweetened gums can be a risk factor for carious lesions since the sugars in dental plaque participate in the cavitation process; sugary gums can be more harmful than hard boiled sucking sweets since they do not melt and can get pressed against teeth for a longer time. The act of chewing, however, promotes the production of saliva, which, in turn, promotes the removal of food particles while neutralizing the acid pH, which follows the sugar intake. These benefits are not enough to counterbalance the negative effects of chewing sugary gums. In fact, few children keep chewing long after the gum has lost its taste, and no kids will continue chewing an old and flavourless piece of gum when there is a fresh one available. The prolonged contact time between immediately accessible new sugars and the oral plaque will promote the development of carious lesions.

Eating more than two pieces of fresh fruit per day was correlated to an increase in the risk of MC. To date, there is no consensus on the cariogenic potential of fruit, and it is not fully recognised whether an excessive daily consumption of fruit is related to caries risk increases [31,32].

Different studies estimated the relative cariogenic potential index of fruits according to their sugar content and texture (viscosity), evaluating the factors that most affect the development of dental caries by measuring the oral pH and acidogenic potential [33]. Some fruits might have a higher cariogenic potential than others, including bananas (for viscosity and sugar content), which are usually present in the children’s diets, and kiwi (for acidic pH) [34]. Another explanation of this result could be attributed to the Hawthorne effect, which could lead to changes in behaviour (in this case, in questionnaire answers) in the presence of observers from whom a certain positive rather than negative response is expected (in this case, the higher amount of fruit consumed).

The frequent weekly consumption of milk, yogurt, and dairy products (greater than two times per week) resulted in a reduction in severe carious lesion development risk for children. This result is probably related to cheese stimulating salivary secretion and the increase in plaque calcium concentration influencing the balance between the demineralisation and remineralisation of enamel [31]. Milk, yogurt, and cheese are important parts of the human diet, and several published reviews have concluded that milk has very low cariogenicity and may have some potential to protect against caries [33]. More recent observational epidemiological studies have reported that milk consumption is associated with a lower incidence of caries. Reasons for these favourable caries-related properties include the lower acidogenicity of lactose compared with other dietary sugars and the protective effects of calcium, phosphate, proteins, and fats [35]. 

The higher prevalence of caries among FPMs is likely to be related to several factors and, mainly, to its morphological characteristics, the early time of its eruption, and its location in the oral cavity [36,37,38,39]. In our study, the prevalence of pit and fissure sealants on the FPMs was negligible. The very low prevalence of dental treatment for carious lesions suggests that children do not receive any treatment and the lesions remain untreated in the face of an imperative and timely need for treatment. In Italy, the essential levels of care cover paediatric dental visits, but parents in deprived contexts are often unaware that they can access free-of-charge oral health care for their children [14,40].

Early carious involvement of FPM may have serious consequences in the prognostics of oral health in adults. Children can present with one or more FPMs affected by severe caries with a poor prognosis because they are no more susceptible to conservative-endodontics. The early extraction of an FPM has several dental and skeletal consequences interrelated to the closure of post-extraction space due to the early eruption of the second permanent molar, to the lingual tipping and retrusion of incisors, and the counter-clockwise rotation of the occlusal plane [41]. 

## 5. Conclusions

This survey gives a picture of widespread dental caries among 6–7 years old children living in Palermo (Sicily, Southern Italy), especially to a moderate or severe degree, with a high concentration in some highly deprived districts. Moreover, a very low prevalence of sealants and restorations was found, suggesting that children do not receive any treatment and the lesions remain untreated in the face of an imperative and timely need for treatment.

Policy makers should implement welfare to support oral health and improve prophylaxis strategies for dental caries in contexts where deprivation is exacerbated by public expenditure cuts. 

## Figures and Tables

**Table 1:** Descriptive statistics of 6–7 years old schoolchildren (*n* = 995), by caries stages and sex.

	Males		Females		Total	
	*n* (%)	M ± SD	*n* (%)	Mean ± SD	*n* (%)	Mean ± SD
Caries-free (ICDAS_0_)	181 (54.4)	18.56 ± 3.53	152 (45.7)	18.56 ± 3.6	333 (100.0)	18.56 ± 3.56
Initial caries (ICDAS_1–2_)	251 (50.4)	0.98 ± 1.37	247 (49.6)	1.06 ± 1.32	498 (100.0)	1.02 ± 1.34
Moderate lesion (ICDAS_3–4_)	234 (53.9)	1.02 ± 1.47	200 (46.1)	1.03 ± 1.57	434 (100.0)	1.03 ± 1.52
Extensive lesion (ICDAS_5–6_)	136 (55.7)	0.73 ± 1.59	108 (44.3)	0.65 ± 1.62	244 (100.0)	0.69 ± 1.61

**Table 2:** Multivariable association of caries stages related to socio-economic variables and diet/behavioural habits.

Variables ^§^		AdjOR	95% CI	*p*-Value ^§§^
Initial caries (ICDAS_1–2_)				
Deprivation index	Most deprived 3rd- 4th- 5th quintiles	1.46	1.07–1.99	0.017
Municipal District *	Second (5th)	1.73	1.19–2.51	0.004
	Third (3rd)	0.57	0.37–0.88	0.011
	Fifth (5th)	2.14	1.5–3.05	<0.001
	Seventh (4th)	2.49	1.61–3.84	<0.002
Moderate lesion (ICDAS_3–4_)				
Mother’s education	High-school or above	0.64	0.44–0.91	0.014
Father’s education	High-school or above	0.65	0.46–0.93	0.018
Chewing gum consumption	Always/Often	1.61	1.03–2.52	0.038
Daily fruits consumption	>2×/day	1.82	1.3–2.55	0.001
Sweet drinks/carbonated drinks consumption	Always/Often	1.63	1.19–2.23	0.002
Dental visits in last year	>2×/year	2.80	1.75–4.48	<0.001
Municipal District *	First (5th)	5.09	1.91–13.62	0.001
	Second (5th)	1.96	1.28–3	0.002
	Fifth (5th)	1.56	1.01–2.41	0.044
	Sixth (5th)	1.65	1.09–2.49	0.017
	Seventh (4th)	2.77	1.64–4.66	<0.001
Extensive lesion (ICDAS_5–6_)				
Father’s education	High-school or above	0.44	0.29–0.66	<0.001
Father’s working status	Unemployed/Homemaker	2.30	1.52–3.47	<0.001
Chewing gum consumption	Always/Often	2.25	1.39–3.65	0.001
Weekly dairy products consumption	≥2×/week	0.63	0.44–0.92	0.015
Dental visits in last year	>2×/year	7.12	4.25–11.94	<0.001
Municipal District *	First (5th)	3.60	1.37–9.41	0.009
	Second (5th)	1.99	1.27–3.12	0.003
	Seventh (4th)	1.82	1.02–3.22	0.041

^§^ Reference categories: Secondary (Schools Mother’s and Father’s education), Seldom or never (Visits to a dentist), No (Cheese), VIII (1st) Municipal District. ^§§^ Adjusted ORs and 95% CIs from ordered logistic regression; only statistically significant coefficients are shown. * The mode of Deprivation Index quintiles is shown in parentheses.

**Table 3:** The occurrence of decayed first permanent molars in 742 (out of 995) 6–7 years old children by caries stages.

0 Teeth	1 Tooth	≥2 Teeth
Initial lesion ICDAS_1–2_		
504 (67.92)	114 (15.36)	124 (16.71)
Moderate lesion ICDAS_3–4_		
656 (88.41)	47 (6.33)	39 (5.26)
Extensive lesion ICDAS_5–6_		
739 (99.60)	3 (0.40)	0 (0.0)

Percentages are shown in parentheses.

**Table 4:** Presence and number of affected surfaces, by caries stages, in all teeth and in first permanent molars.

Stages of Caries and Surfaces	First Permanent Molars Mean ± SD	Total Sample Mean ± SD	*p*-Value
Initial lesion ICDAS_1–2_	0.85 ± 1.49	1.08 ± 1.43	<0.01
Occlusal	0.54 ± 0.93	0.93 ± 1.31	0.01
Non-occlusal	0.31 ± 0.63	0.15 ± 0.52	0.13
Moderate lesion ICDAS_3–4_	0.27 ± 0.88	1.21 ± 1.86	<0.01
Occlusal	0.18 ± 0.56	0.67 ± 1.22	0.01
Non-occlusal	0.09 ± 0.38	0.53 ± 1.1	<0.01
Extensive lesion ICDAS_5–6_	0.01 ± 0.11	1.57 ± 4.63	<0.01
Occlusal	0.00 ± 0.06	0.48 ± 1.32	0.01
Non-occlusal	0.01 ± 0.05	1.09 ± 3.46	<0.01

**Table 5:** Multivariable association of caries stages of first permanent molars related to socio-economic variables and diet/behavioural habits.

Variables ^§^		AdjOR	95% CI	*p*-Value
Initial lesion (ICDAS_1–2_)				
Municipal District	Second	1.87	(1.14–3.04)	0.012
	Third	0.29	(0.14–0.62)	0.001
	Fifth	2.07	(1.30–3.29)	0.002
	Sixth	0.35	(0.19–0.66)	0.001
	Seventh	2.33	(1.32–4.10)	0.003
Mother’s working status	Unemployed/Homemaker	1.51	(1.03–2.20)	0.034
Deprivation index	Most deprived (3rd- 4th- 5th quintiles)	1.70	(1.07–2.68)	0.024
Moderate lesion (ICDAS_3–4_)				
Father’s education	High-school or above	0.48	(0.26–0.86)	0.013
Municipal District	Seventh	5.26	(2.74–10.10)	<0.001

^§^ Reference categories: Mandatory (Mother’s and Father’s education), Seldom or never (Visits to a dentist), No (Cheese), VIII (Municipal District).

## Data Availability

The data presented in this study are available on request from the corresponding author.

## Supplementary Materials

The following supporting information can be downloaded at: https://www.mdpi.com/article/10.3390/jcm12134343/s1, Table S1: Univariable association of caries staging related to demographic and socio-economic determinants; Table S2: Caries severity levels (ICDAS) association to diet, behavioural habits and clinical status of the first permanent molar.
