# Supplementary material for: Caries Severity, Decayed First Permanent Molars and Associated Factors in 6–7 Years Old Schoolchildren Living in Palermo (Southern Italy)
Source: J Clin Med. 2023 Jun 28;12(13):4343. doi: 10.3390/jcm12134343 (PMC10342988; doi:10.3390/jcm12134343)
Supplement: Supplementary file 1 [file jcm-12-04343-s001.zip › jcm-2398864-supplementary.pdf]

Supplementary Materials

Table S1. Univariable association of caries staging related to demographic and socio-economic determinants.

		**Total**		**Caries-free ($ICDAS_0$)**	**Initial lesion ($ICDAS_{1-2}$)**	**Moderate lesion ($ICDAS_{3-4}$)**	**Extensive lesion ($ICDAS_{5-6}$)**
**Variables**		*n*	%	*mean±SD*	*mean±SD*	*mean±SD*	*mean±SD*
**Gender**	Males	523	52.56	18.56±3.53	0.98±1.37	1.02±1.47	0.73±1.59
	Females	472	47.44	18.56±3.60	1.06±1.32	1.03±1.57	0.65±1.62
	p-value		*0.106*	0.947	0.257	0.721	0.511
**Mother's marital status**	in couple	*887*	*90.32*	18.57±3.55	1.00±1.33	1.03±1.52	0.69±1.60
	not in couple	95	9.74	18.52±3.57	1.14±1.46	1.01±1.43	0.69±1.71
	p-value		***<0.001***	0.197	0.635	0.944	0.388
**Father's marital status**	in couple	879	91.46	18.59±3.54	1.01±1.34	1.03±1.53	0.68±1.56
	not in couple	*82*	*8.53*	18.51±3.57	1.13±1.47	1.05±1.45	0.67±1.73
	p-value		***<0.001***	*0.21*	*0.72*	*0.74*	*0.16*
**Mother's education**	Middle school or below	433	44.0	17.52±3.85	1.13±1.39	1.49±1.7	1.12±2.05
	High school or above	551	55.99	19.41±3.06	0.93±1.30	0.66±1.23	0.34 ± 1.01
	p-value		***<0.001***	< 0.001	0.027	< 0.001	< 0.001
**Father's education**	Middle school or below	*486*	*50.47*	17.74±3.81	1.10±1.37	1.39±1.68	1.03±1.92
	High school or above	477	49.53	19.44±3.02	0.94±1.34	0.66±1.23	0.31±0.98
	p-value		***0.466***	< 0.001	0.055	< 0.001	< 0.001
**Mother's working status**	Employed	352	35.81	19.46±3.03	0.92±1.33	0.66±1.29	0.39±1.13
	Unemployed/Homemaker	*631*	*64.19*	18.08±3.71	1.06±1.35	1.22±1.58	0.86±1.8
	p-value		***<0.001***	< 0.001	0.022	< 0.001	< 0.001
**Father's working status**	Employed	736	77.31	18.97±3.33	0.96±1.30	0.87±1.42	0.5±1.35
	Unemployed/Homemaker	216	22.69	17.31±3.85	1.21±1.50	1.5±1.68	1.25±2.04
	p-value		***<0.001***	< 0.001	0.075	< 0.001	< 0.001
**Deprivation index**	Least deprived (1st- 2nd quintiles)	*270*	*27.13*	19.77±3.01	0.88±1.40	0.56±1.10	0.31±1.16
	Most deprived (3rd- 4th- 5th)	725	72.86	18.11±3.65	1.07±1.32	1.20±1.61	0.83±1.72
	p-value		***<0.001***	< 0.001	0.002	< 0.001	< 0.001
**Municipal District***	First (5th)	28	2.81	15.79±4.53	1.36±1.37	2.14±1.72	2.14±3.24
	Second (5th)	*146*	*14.67*	18.28±3.56	1.25±1.32	1.27±1.71	0.82±1.51
	Third (3rd)	125	12.56	18.9±3.16	0.56±0.95	0.86±1.42	0.56±1.27
	Fourth (1st)	126	12.66	19.37±3.66	1.02±1.54	0.75±1.43	0.33±0.93
	Fifth (5th)	*148*	*14.87*	18.27±3.56	1.47±1.64	0.98±1.37	0.77±1.73
	Sixth (5th)	174	17.49	18.51±3.21	0.69±1.11	0.99±1.43	0.57±1.41
	Seventh (4th)	92	9.25	16.74±3.66	1.39±1.21	1.92±1.8	1.26±1.94
	Eighth (1st)	*156*	*15.67*	19.79±3.20	0.83±1.25	0.53±1.10	0.42±1.58
	p-value		***<0.001***	***<0.001***	***<0.001***	***<0.001***	***<0.001***

* The mode of Deprivation Index quintiles is shown between parentheses

Table S2. Caries severity levels (ICDAS) association to diet, behavioural habits and clinical status of the first permanent molar.

		Total		Caries-free ($ICDAS_0$)	Initial lesion ($ICDAS_{1-2}$)	Moderate lesion ($ICDAS_{3-4}$)	Extensive lesion ($ICDAS_{5-6}$)
**Variables**		**n**	**%**	**mean±SD**	**mean±SD**	**mean±SD**	**mean±SD**
Daily toothbrushing habit	Seldom/Never	218	22.09	17.91±3.83	1.09±1.42	1.32±1.65	0.88±1.79
	After meals	769	77.91	18.74±3.47	1.00±1.33	0.94±1.46	0.64±1.55
	p-value		***<0.001***	*0.048*	*0.664*	*0.006*	*0.032*
PFM affected by caries	No	731	73.47	19.21±3.31	0.54±0.91	0.74±1.31	0.47±1.31
	Yes	264	26.53	16.75±3.64	2.34±1.47	1.81±1.75	1.30±2.11
	p-value		***<0.001***	***<0.001***	***<0.001***	***<0.001***	***<0.001***
Orthodontic devices	No/Former	966	99.18	18.55±3.57	1.03±1.35	1.02±1.51	0.70±1.62
	Current	8	0.82	18.88±4.45	0.63±1.06	1.38±2.07	0.75±1.39
	p-value		***<0.001***	*0.512*	*0.652*	*0.487*	*0.680*
Additional fluoride products use	No	750	76.53	18.34±3.64	1.02±1.37	1.08±1.52	0.74±1.65
	Yes	230	23.47	19.33±3.16	1.03±1.29	0.80±1.45	0.47±1.35
	p-value		***<0.001***	*0.012*	*0.759*	*0.002*	*0.052*
Chewing-gum consumption	Seldom/Never	857	87.63	18.82±3.41	1.01±1.36	0.91±1.44	0.59±1.45
	Always/Often	121	12.37	16.78±4.05	1.05±1.20	1.74±1.78	1.43±2.35
	p-value		***<0.001***	*< 0.001*	*0.173*	*<0.001*	*<0.001*
Daily fruit consumption	≤ 2x/day	725	77.37	18.83±3.43	0.99±1.34	0.92±1.45	0.58±1.43
	> 2x/day	212	22.62	17.74±3.85	1.15±1.41	1.38±1.65	0.96±1.97
	p-value		***<0.001***	*0.001*	*0.148*	*< 0.001*	*0.008*
Weekly dairy product consumption	< 2x/week	397	40.72	18.03±3.76	1.08±1.43	1.16±1.62	0.93±1.84
	≥ 2x/week	578	59.28	18.93±3.39	0.97±1.28	0.92±1.42	0.53±1.41
	p-value		***<0.001***	*0.015*	*0.719*	*0.081*	*<0.001*
Sugary drinks/sweetened pacifier use before bedtime	Seldom/Never	880	94.52	18.81±3.38	1.01±1.34	0.95±1.47	0.57±1.36
	Always/Often	51	5.47	16.39±4.57	1.04±1.52	1.59±1.71	1.88±3.22
	p-value		***<0.001***	*0.001*	*0.679*	*0.005*	*0.007*
Eating snacks between meals	Seldom/Never	649	66.63	18.71±3.52	1.06±1.37	0.98±1.52	0.60±1.49
	Always/Often	325	33.37	18.23±3.64	0.94±1.30	1.07±1.53	0.86±1.81
	p-value		***<0.001***	*0.08*	*0.28*	*0.48*	*<0.01*
Sweet drinks/carbonated drinks consumption	Seldom/Never	667	68.06	18.97±3.24	1.02±1.35	0.86±1.44	0.51±1.33
	Always/Often	313	31.94	17.77±4.05	1.02±1.33	1.32±1.59	1.07±2.04
	p-value		***<0.001***	*0.001*	*0.935*	*<0.001*	*<0.001*
Dental visit in the previous year	≤ 2x/year	888	90.89	18.82±3.39	0.99±1.33	0.91±1.43	0.57±1.50
	> 2x/year	89	9.11	16.55±4.18	1.25±1.44	1.87±1.93	1.64±1.93
	p-value		***<0.001***	*0.001*	*0.056*	*<0.001*	*<0.001*

PFM: Primary First Molar
